# Giant virus vs amoeba: fight for supremacy

**DOI:** 10.1186/s12985-019-1244-3

**Published:** 2019-11-04

**Authors:** Graziele Oliveira, Bernard La Scola, Jônatas Abrahão

**Affiliations:** 1 Laboratório de Vírus, Instituto de Ciências Biológicas, Departamento de Microbiologia, Universidade Federal de Minas Gerais, Belo Horizonte, MG 31270-901 Brazil; 2 Microbes, Evolution, Phylogeny and Infection (MEPHI), Aix-Marseille Université UM63, Institut de Recherche pour le Développement IRD 198, Assistance Publique, Hôpitaux de Marseille (AP-HM), Marseille, France; 3 Institut Hospitalo-Universitaire (IHU)-Méditerranée Infection, Marseille, France

**Keywords:** Giant virus–host interactions, Marseillevirus, Giant vesicles, Mimivirus, Cheshire cat, Tupanvirus dissemination, Faustovirus mariensis, Antiviral mechanism, Virophage

## Abstract

Since the discovery of mimivirus, numerous giant viruses associated with free-living amoebae have been described. The genome of giant viruses can be more than 2.5 megabases, and virus particles can exceed the size of many bacteria. The unexpected characteristics of these viruses have made them intriguing research targets and, as a result, studies focusing on their interactions with their amoeba host have gained increased attention. Studies have shown that giant viruses can establish host–pathogen interactions, which have not been previously demonstrated, including the unprecedented interaction with a new group of small viruses, called virophages, that parasitize their viral factories. In this brief review, we present recent advances in virophage–giant virus–host interactions and highlight selected studies involving interactions between giant viruses and amoebae. These unprecedented interactions involve the giant viruses mimivirus, marseillevirus, tupanviruses and faustovirus, all of which modulate the amoeba environment, affecting both their replication and their spread to new hosts.

## Background

In 2003, virologists were surprised by the discovery of the first giant virus of amoeba, which researchers named mimivirus [1]. The discovery of mimivirus has shed light on new approaches for virus isolation and has led to an increase in the number of giant virus isolates [2–14]. Years later, small viruses infecting the viral factories (VFs) of giant viruses were discovered. These viruses were named virophages and they revealed new dimensions of the interactions existing among giant viruses [15]. Some of the main hosts associated with the giant viruses described are the amoebas of the genus *Acanthamoeba*. These amoebas, besides being associated with human diseases, play a relevant role in ecosystems, acting both as predators and hosts for microorganisms [16–21]. In addition to the acanthamoebas*, Vermamoeba vermiformis*, another species of free-living amoeba, has been described as one of the hosts of giant viruses, such as tupanvirus, faustovirus and kaumoebavirus [8, 11, 14, 22]. These protozoans obtain their nutrients through phagocytosis. This process is one of the ways in which many giant viruses, such as mimivirus, initiate their replication cycles [23–25]. Characterization of giant viruses has revealed unimaginable genomic complexity, including the existence of hundreds of genes associated with activities that have never before been attributed to viruses. Here, we examine the discoveries related to virophage–giant virus–host interactions and highlight selected studies that have investigated the interactions between host amoebas and the giant viruses mimivirus, marseillevirus, tupanviruses and faustovirus mariensis.

## Main text

### Mimivirus and the ‘Cheshire cat’ theory

**Fig. 1:**
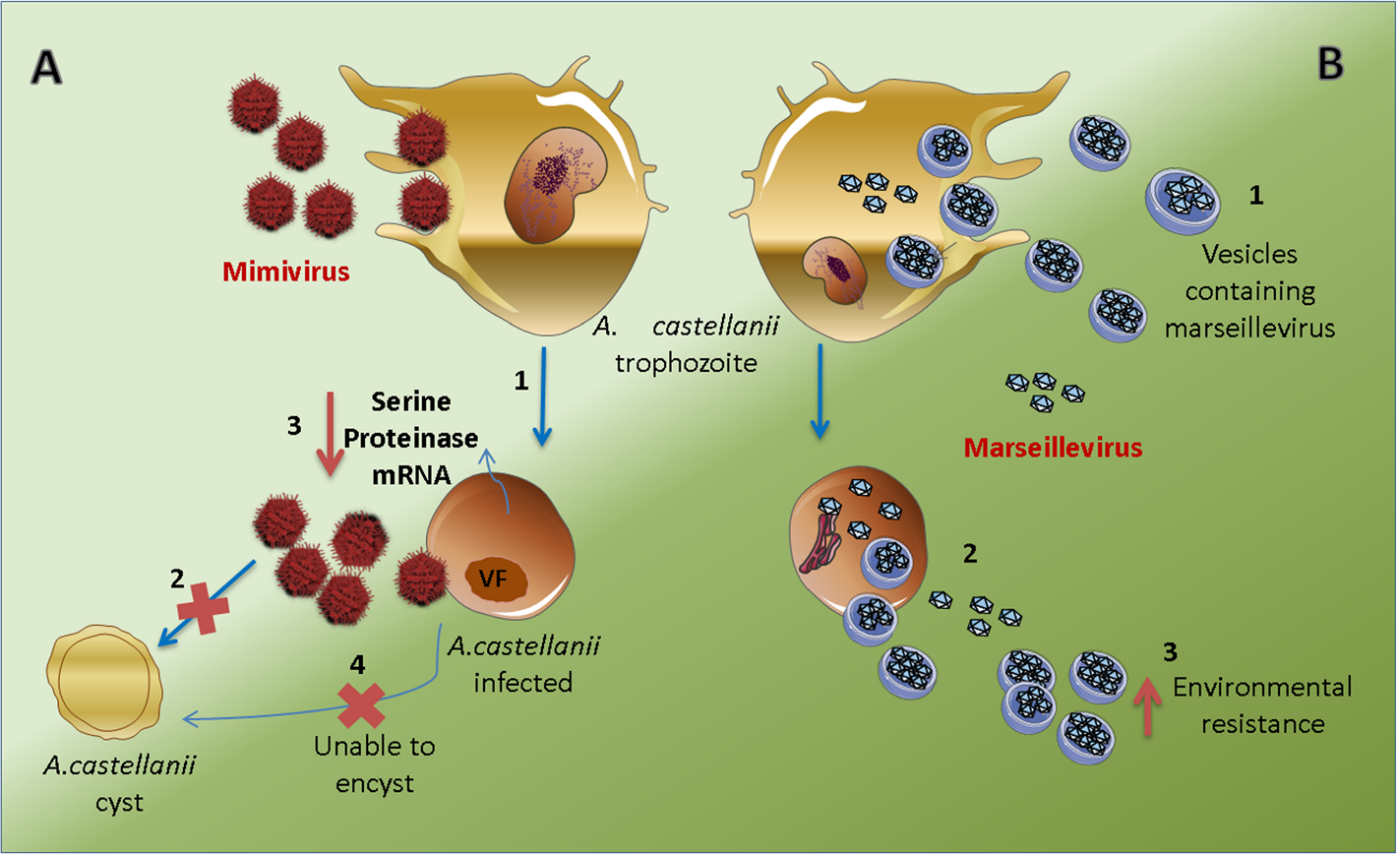
Interaction between mimivirus and marseillevirus and their host *Acanthamoeba*. **a** Mimivirus is able to infect and establish productive replication in *A. castellanii* trophozoites (1), but it is unable to infect cysts (2). When *A. castellanii* is infected by mimivirus, the expression of a serine proteinase gene is blocked (3), the encystment is hampered (4), and the infection occurs. **b** Vesicles containing marseilleviruses particles trigger phagocytosis in *A. castellanii* (1) since they fulfill the > 500 nm size requirement. Productive infection occurs and the particles may be released as individual particles or in vesicles (2). Vesicles promote infectivity and increase environmental resistance compared to single particles (3). Viral factory: VF

The mimiviruses were the first amoeba-infecting giant viruses to be discovered, which subsequently led to the formation of the *Mimiviridae* family. Acanthamoeba polyphaga mimivirus (APMV) (also known as mimivirus) was the first isolate and, as such, has become the prototype species of the *Mimivirus* genus [1, 26]. Currently, numerous mimivirus isolates have been found from some of the most diverse environments associated with amoeba of the *Acanthamoeba* genus, the main known host of mimivirus [1, 27–29]. The ‘Cheshire Cat’ escape strategy is a phenomenon previously described between a unicellular eukaryote, *Emiliana huxleyi*, and emiliania huxleyi virus, a phycodnavirus. *Emiliania huxleyi* has two stages in its life cycle, a haploid, non-calcified phase and a diploid, calcified phase [30]. Researchers have demonstrated that only diploid-phase cells can be infected by emiliania huxleyi virus, in contrast to the haploid phase, which is resistant to infection. Moreover, exposure of the diploid phase of *Emiliania huxleyi* to phycodnavirus induces the transition of neighboring cells to the haploid phase [30]. *Acanthamoeba* undergoes two life cycle stages (trophozoite and cyst), and APMV is unable to infect cysts. On the other hand, it has been shown that when trophozoites are infected, the viral progeny titer increases about 2.5 logs (500-fold) 24 h post infection, and an evident cytopathic effect (CPE) is observed [31, 32]. The encystment process involves a high level of cellular and molecular regulation, induced by signals such as osmotic stress, starvation, and temperature [33–36]. Previous studies have shown that the cytoskeleton, as well as serine proteases and other factors, play a crucial role in the encystment process [37–40]. A serine-type proteinase called encystment-mediating subtilisin-like serine proteinase (EMSP) has been associated with the encystment process in *Acanthamoeba.* Previous work has demonstrated that mimivirus infection reduces both mRNA and protein levels of this serine proteinase in *Acanthamoeba castellanii*. Furthermore, the virus was able to prevent the expression of EMSP when infected cells were added to an encystment saline solution [31]. It has not yet been described how the mimivirus is able to reduce the expression of EMSP. It has been shown that the inhibition of serine-proteinase genes negatively affects the encystment. Analysis of the mimivirus-expressed genes associated with the data obtained in this study suggested that the gene R700, present in the APMV genome, which encodes a serine protease inhibitor, might be one of the genes involved in the down regulation of this process [32]. Other proteins may act in the regulation of the encystment process in *Acanthamoeba castellanii* infected by mimivirus, and further investigation will be necessary to better understand the roles of these protease inhibitors. This study suggested that the encystment process can allow *Acanthamoeba* populations to escape mimivirus infections; however, mimivirus has the ability to respond to this evasion tactic by preventing the encystment process (Fig. 1a). This study was one of the first to investigate a type of interaction between giant viruses and their host, with respect to modulation of the host life cycle.

Consideration of the aforementioned study led Silva and collaborators, in 2016, to propose that the ‘Cheshire Cat’ theory could be extended to describe relationships between mimivirus and its hosts [30, 32]. Parallels can be drawn between findings related to infection of *Acanthamoeba* by mimivirus and infection of *Emiliana huxleyi* by emiliana huxleyi virus. First, both hosts undergo two life stages. Similar to *Emiliana huxleyi*, which can only be infected during the diploid phase of its life cycle, mimivirus is able to infect only the trophozoite stage of the *Acanthamoeba* life cycle, while cysts are resistant to infection (Fig. 1a). Moreover, it has been shown that during APMV infection a small percentage of acanthamoeba cells are able to encyst [30–32]. There is a gap in the literature when it comes to amoebal communication and associated factors. As a result, there remains a rich supply of research opportunities in the investigation of giant virus–host interactions.

### Viral megalomania: the marseilleviruses and their giant infectious vesicles

Marseilleviruses was the second group of amoebal giant viruses to be discovered. The first marseillevirus was isolated in *Acanthamoeba castellanii* cells inoculated with a water sample collected from a cooling tower in Paris, France [2]. This virus was named marseillevirus marseillevirus (MsV), and many other marseillevirus-like viruses have been described since. They have been isolated in France, as well as other countries, including Tunisia, Senegal, Australia, Japan, Malaysia, India, and Brazil [2, 41–48]. Researchers have demonstrated that the genome of MsV is approximately 400 kb and is composed of many genes apparently obtained from hosts and their parasites or symbionts. Based on these and other findings, it was proposed that amoebae are like ‘melting pots,’ where giant viruses containing complex gene repertoires of various origins can emerge [2]. Phagocytosis is the process by which most of the giant viruses can initiate their replication cycles in amoebas [1, 2, 4, 6, 7, 25, 49]. However, for the phagocytosis process to be triggered, particles must be > 500 nm so that they can be recognized [50]. MsV has an icosahedral particle, with a diameter of about 250 nm, surrounded by 12-nm-long surface fibers [2]. Curiously, although MsV does not reach the prerequisite size for phagocytosis, this virus is still able to successfully replicate in *Acanthamoeba*, suggesting that there may be a different mechanism of interaction between MsV and its host, functioning to initiate the viral cycle.

Looking for answers about marseillevirus and host interactions, in 2016 Arantes and collaborators performed a detailed study of the MsV replication cycle and unexpectedly discovered that marseillevirus is able to produce and release giant vesicles that can contain > 1000 viral particles. The vesicles varied in terms of size (300 nm to 1000 nm) and number of membranes. Immunofluorescence and immunoblotting assays targeting the endoplasmic reticulum (ER), Golgi complex, and endosome revealed that the membranes of the vesicles originate from the ER, while the MsV internal membrane seems to be derived from the amoebal endosome [51, 52].

Questions remained regarding whether the giant vesicles could allow for phagocytosis. This prompted research demonstrating that such giant vesicles of MsV are able to trigger the phagocytosis process as a result of their large size, which makes recognition possible (Fig. 1b). This new mechanism of viral entry highlights a remarkable adaptation of marseillevirus to the amoeba lifestyle since phagocytosis is one of the main physiological processes related to amoebal feeding. Remarkably, in addition to entry mediated by giant-vesicle-induced phagocytosis, the entry of MsV may also occur by the phagocytosis of aggregated particles and by acidification-dependent endocytosis of single particles [51]. This work revealed that these giant infective vesicles are some of the main ways by which MsV successfully initiates its replication cycle, revealing a host–virus interaction that has not been previously described among DNA viruses.

In addition to the fact that many approaches have demonstrated the role of vesicles in the biology of MsV and the maintenance of these viruses in nature, it was also shown that the giant vesicles can contain one or several membranes. Therefore, it was predicted that the number of membranes within vesicles can influence the entry of MsV into host amoeba. It was suggested that vesicles containing only one membrane merge with the phagosome membrane and release their particles inside the cytoplasm of the amoeba, while the outer membrane merges with the phagosome and the inner vesicle is released in cases where vesicles contain several membranes [51]. Further investigation will be required for researchers to fully elucidate the uncoating process employed by marseillevirus particles.

Since it has been suggested that MsV particles may be released from the host amoeba within vesicles, the hypothesis that the vesicles could generate an adaptive advantage for MsV was tested. It has been demonstrated that the dispersion of some RNA viruses by vesicles is an act used to escape from the host immune system, providing an adaptive advantage [53, 54]. Although the presence of an adaptive immune system in the MsV host amoeba has not been shown, we cannot rule out the possibility that the virus is capable of utilizing vesicles in a similar manner, especially since marseillevirus has already been associated with humans, which have a complex immune system. However, more studies need to be conducted on this topic [55–57]. Considering that MsV is often isolated from environmental samples, it has been suggested that vesicles may be relevant for the maintenance of this virus in the environment. This happens because vesicles initiate the viral replication cycle more quickly than single particles. In addition, when giant vesicles and isolated MsV particles were exposed to extreme heat (70 °C), it was observed that the vesicles conferred a longer duration of temperature resistance to the virus than what exists for single viral particles. Thus, giant vesicles could confer resistance to MsV against environmental factors, in addition to promoting greater efficiency of infection, facilitating the spread of the virus to other susceptible cells and enabling phagocytosis of the virus (Fig. 1b). Finally, the possibility was raised that infection through vesicles evolved as a powerful mechanism to boost the replicative success of this virus within its natural hosts and/or its survival in the environment.

### Tupanvirus: an unexpected structural and genomic complexity

Among the many new giant viruses that have been discovered in recent years, tupanvirus has drawn our attention, not only due to its genomic and structural characteristics that distinguish it from all other described viruses, but also because of its ability to establish interactions that have never been demonstrated among giant viruses. Tupanviruses were isolated in Brazil from the Pantanal soda lake region and in deep ocean sediments collected at a depth of 3000 m in the region of Campos dos Goytacazes. Tupanvirus particle sizes vary from 1.2 μm to 2.5 μm, and they are composed of a ~ 450-nm capsid covered by fibrils with a vertex modified in a starfish shape [14]. Among its most noteworthy morphological features is the presence of a long tail attached to the capsid, measuring ~ 550 nm [14]. The tupanviruses have one of the largest genomes among mimiviruses members, which is composed of linear, double-stranded DNA of ~ 1,5 Mb coding more than 1250 genes. The genes in the genome of the tupanvirus that were the most surprising were those related to translation machinery, including 20 aminoacyl tRNA synthetases and 70 tRNA, in addition to other factors associated with translation and tRNA/mRNA maturation and modification of ribosome proteins [14]. As if all the novelties related to the discovery of tupanvirus were not enough, it was shown that, unlike other giant viruses, tupanvirus is able to infect a wide range of hosts. In addition, the study of the interaction between tupanvirus and host showed that tupanvirus is able to trigger a host ribosomal shutdown [14]. A recent study described a virus–host interaction in which tupanvirus-infected amoebas were induced to aggregate to uninfected cells, forming bunches that seemed to be important for tupanvirus fitness [58]. In the following two sections, we will review these interactions described for tupanvirus.

#### The broad host range of tupanvirus and host ribosomal shutdown

**Fig. 2:**
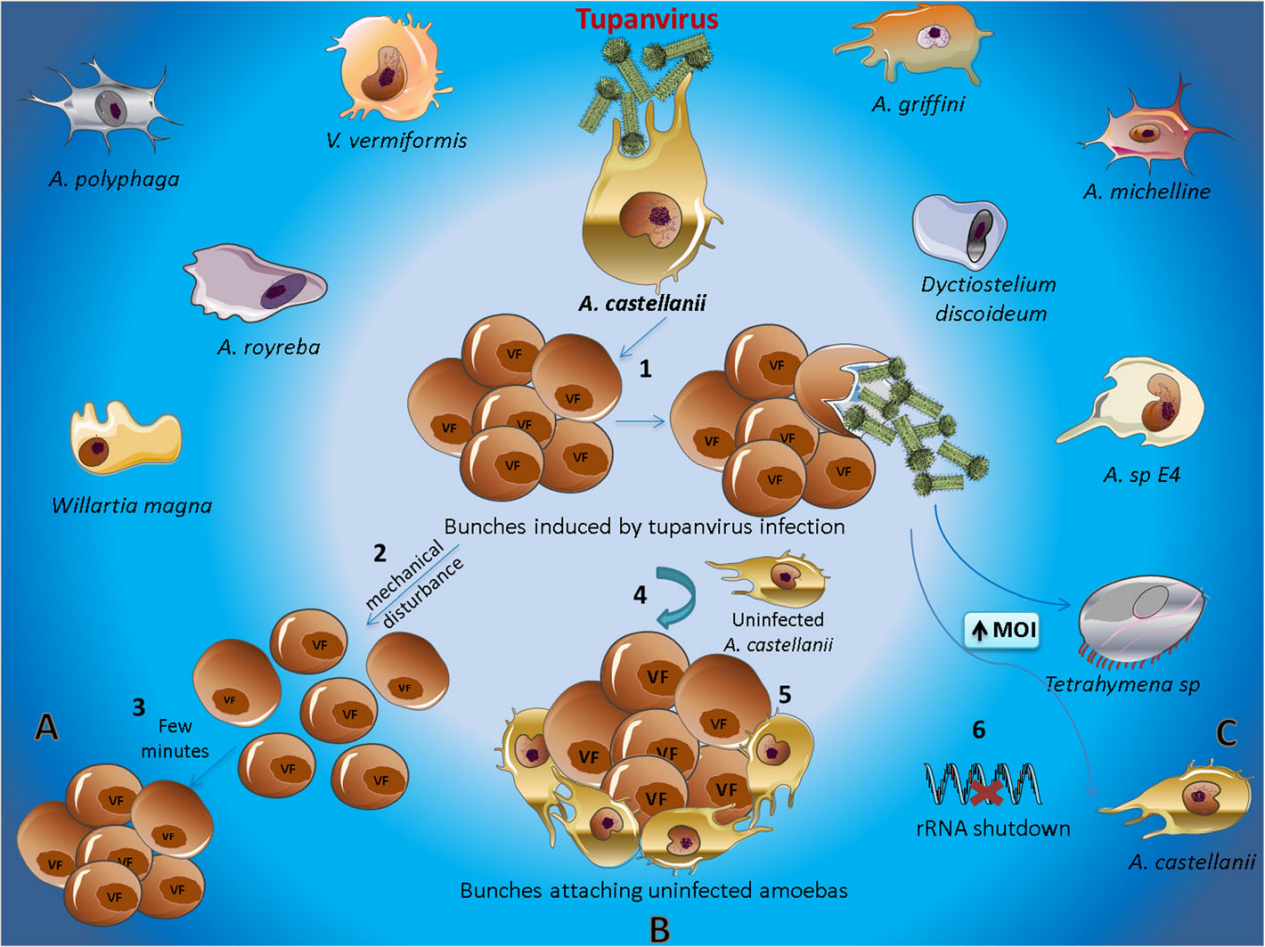
Host–tupanvirus interactions*. A. castellanii* infection by tupanvirus induces the formation of bunches (1). **a** Mechanical disturbances can disrupt (2) the bunches, which are able to reform a few minutes after mechanical separation (3). **b** Uninfected *A. castellanii* interacting with bunches (4) and being carried by them (5). **c** Tupanvirus causing ribosomal shutdown in *Tetrahymena hyperangularis* and *A. castellanii* at high multiplicity of infection (MOI) (6). Other amoebae in which tupanvirus is able to establish interactions include *A. castellanii*, *A. polyphaga*, *A. sp E4*, *A. griffini*, *V. vermiformis*, *Dyctiostelium discoideum*, *Willartia magna*, *A. michelline*, and *A. royreba* are represented evidencing their broad host range. Viral factory: VF

A differential characteristic of tupanviruses when compared to the other giant viruses is their broad host range. While most of the giant viruses, such as cedratvirus, marseilleviruses, mollivirus, pandoraviruses, mimivirus, faustovirus and kaumoebavirus are able to replicate in only a single known genus of amoeba, the tupanviruses are able to infect a broad host range, such as *A. castellanii, A. polyphaga, A. sp E4, A. griffini, V. vermiformis, Dyctiostelium discoideum*, and *Willartia magna* (Fig. 2) [4–6, 8, 10, 11, 14]. Tupanviruses exhibit CPE and genome replication, but there is no particle burden in *A. michelline* and *A. royreba*. In addition, though tupanviruses are not able to replicate in *Tetrahymena hyperangularis,* the virus is successfully phagocytized and contents consisting of tail and capsid components are released into the cytoplasm of the protozoa. This release triggers a cytotoxic profile characterized by loss of motility, an increase in vacuolization, a large amount of extracellular vesicles, a decrease in the phagocytosis rate, and unexpected ribosomal shutdown (Fig. 2c). The absence of ribosomal subunits in electrophoresis analysis suggested the occurrence of ribosomal degradation. This absence was also observed in *A. castellanii* in experiments in which a high multiplicity of infection (MOI) was used (Fig. 2c). The first hypothesized explanation of the absence of ribosomal subunits was the process of ribophagy, an autophagy process responsible for the degradation of ribosomes in prolonged periods of nutrient deprivation [59]. Analysis of typical ribophagy markers, such as double membrane formation, autophagosome acidification, and examination of ribophagy-related genes, suggested that the ribophagy process may not be the explanation for the shutdown of RNA caused by tupanvirus infection [14, 59]. Nonetheless, ribosomal shutdown does occur as a result of tupanvirus infection, a phenomenon that remains unexplained. Research has provided some clues in pursuit of a plausible explanation. For instance, there may be the presence of an unknown factor, such as a viral protein, carried by the tupanvirus particle. Since ribosomal shutdown is independent of tupanvirus replication, occurring in the presence of inactivated particles by ultraviolet light, but not by particles inactivated by heat. In addition, it was demonstrated that tupanvirus induces host nuclear degradation, providing another possible mechanism for achieving this response since the nucleolus is involved in ribosome biogenesis [14, 60].

### Tupanvirus and its giant bunches: ‘like zombies’ tupanvirus-infected amoebas are induced to aggregate to uninfected cells

Tupanviruses exhibit a CPE that is characterized by amoebae aggregates called bunches. This peculiar CPE led Oliveira and collaborators to investigate the possible biological factors involved in the formation of the bunches induced by tupanvirus. This investigation resulted in the characterization of a new kind of virus–host interaction by tupanvirus. In order to investigate the interaction between tupanvirus and its host in relation to the formation of bunches, initially the authors focused on the characterization of CPE triggered by tupanvirus in the amoeba *A. castellanii* [58]. It has been shown that the effect starts in a manner similar to that described for other giant viruses, such as APMV*,* in which the amoeba becomes rounded. However, unlike that of the other giant viruses, the formation of early bunches can be observed, and they gradually become larger until almost all cells are incorporated into giant bunches [58].

In addition, immunofluorescence assays and electron microscopy analyses showed that bunches are formed by infected and non-infected (or under different infection stage) cells. Another peculiar observation regarding bunches is that the structures are easily disaggregated, either by vortexing or pipetting. However, it was shown that the early bunches are able to re-form a few minutes after mechanical separation, in contrast to late bunches (Fig. 2a). The lack of bunch re-formation indicates that the cells are already dead. This was confirmed experimentally by demonstrating that amoeba in this stage exhibit plasmatic membranes that are almost completely degraded [58].

After the initial characterization of CPE, was investigated a possible factor that may interfere with bunch formation and the possible biological relevance of the bunches promoted by tupanvirus infection. It was observed that during its replication cycle, tupanvirus is able to express a gene coding a mannose-binding protein (MBP) [58]. This protein was previously associated with adhesion in the amoebae *A. castellanii*, where it was shown that the use of mannose functioned to inhibit the adhesion of *A. castellanii* to surfaces [61–66]. MBP contains a three-fold internal repeat domain, and a previous study was able to show that a QXDXNXVXY motif sequence is involved in mannose recognition, highlighting QDN/Y amino acids as essential for the MBP–mannose interaction [67]. Based on these data, we investigated the effect of mannose on the formation of bunches and its biological implications.

Initially, the analyses of MBP on gene expression showed that during the earlier stages of tupanvirus infection the expression levels of cellular MBP transcripts increased significantly, suggesting that cellular MBP gene expression induced by tupanvirus occurs before bunch formation. In addition, a gradual increase (or accumulation) of MBP mRNAs encoded by tupanvirus was observed. Taken together, these data suggested the possible relevance of this gene in the viral replication cycle since the expression of viral and cellular MBP genes is induced during tupanvirus infection. Was also observed that free mannose negatively affected the expression of both the cellular and tupanvirus MBP gene, and when free mannose was added to the culture medium there was an inhibition of bunch formation in a dose-dependent way. Taken together, these data indicated that amoebal bunch formation correlates with viral and cellular mannose receptor gene expression [58].

It was suggested that MBP gene expression induced by tupanvirus may be important for optimizing the formation of bunches. Previous studies have shown that amoeba MBP is itself a glycoprotein containing mannose, which indicates that the interaction between amoebas may occur through interactions between their surface MBP receptors [65]. This assertion is further supported by the observation that the inhibition of MBP expression decreases the potential for interaction among the amoeba, affecting bunch formation [58]. A recent study showed that tupanvirus induces cell aggregation in *V. vermiformis*, which suggests that a similar mechanism may occur during infection in this host. However, further studies will be needed to confirm this hypothesis [22]. Was observed that the bunches are composed of amoebae at different stages of infection, an observation which led to the investigation of whether the bunches were able to interact with uninfected cells. Using biological assays as well as scanning electron microscopy and immunofluorescence analysis, was observed that when the amoeba bunches were brought into contact with uninfected amoebas they were able to interact and hijack uninfected cells (Fig. 2b) [58].

The interaction with uninfected amoebas promoted by the formation of bunches may optimize viral fitness through improving the probability that viral progeny will find a new host cell. Benefits resulting from this adaptation are especially important when considering the diluting effect present in aquatic environments. This adaptation could play an interesting ecological role since keeping uninfected host cells close to amoebas containing a lot of viral particles could facilitate encounters between viral particles and host cells. Therefore, tupanvirus-infected cells act like “zombies,” attaching themselves to uninfected cells and improving the chances of recently formed viral progeny finding a new host cell in which they can propagate.

### *Vermamoeba vermiformis* trapping the enemy faustovirus mariensis

**Fig. 3:**
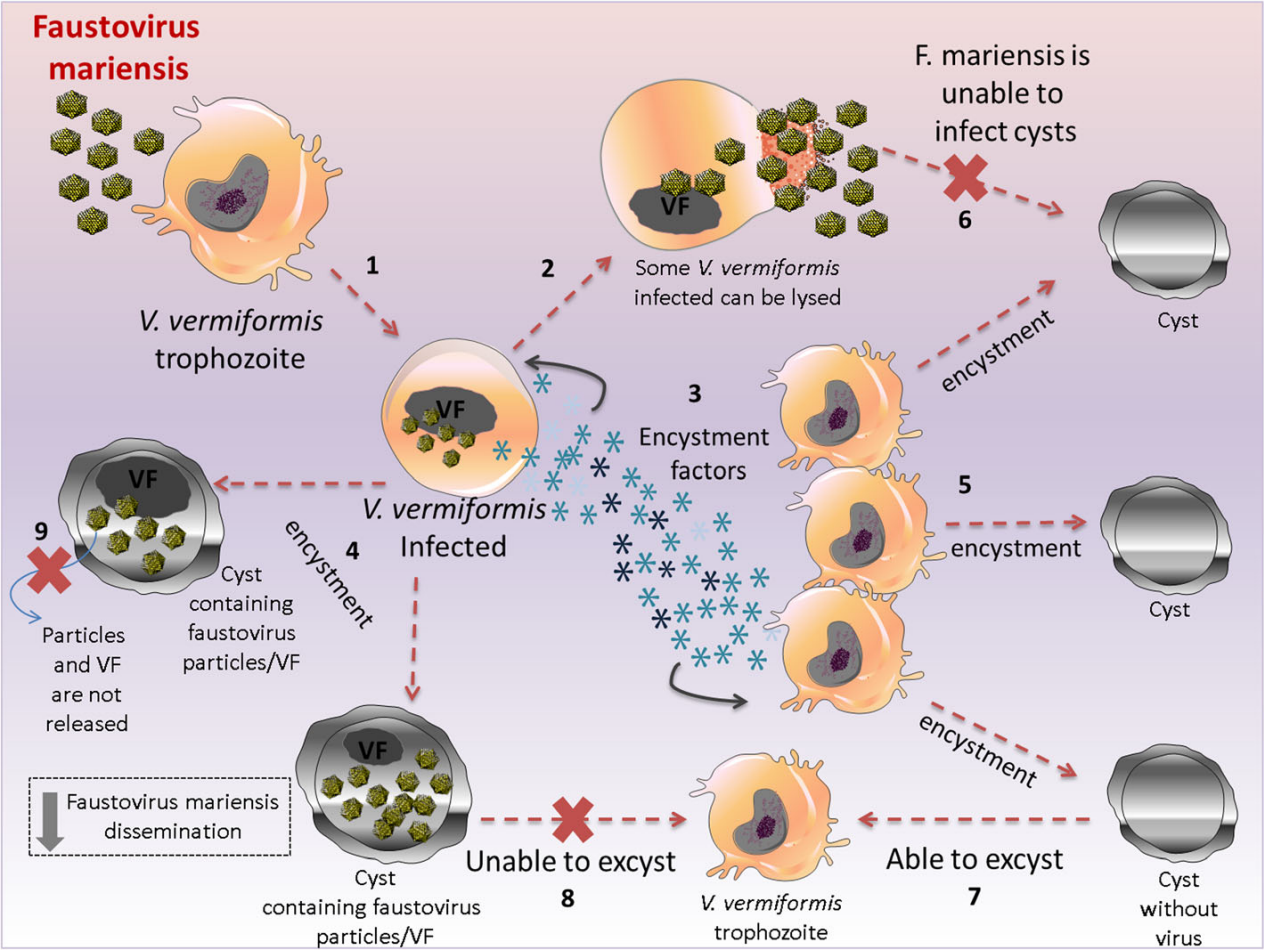
Faustovirus mariensis and *Vermamoeba vermiformis* interactions*.* Faustovirus mariensis is able to infect *V. vermiformis* trophozoites (1), and *V. vermiformis* infected cells can be lysed (2). However, infected cells release encystment factors (3) that trigger the encystment of the infected (4) and uninfected neighbor cells (5), which, in turn, will not be infected since faustovirus mariensis is unable to infect cysts (6). Infected trophozoites are converted to cysts containing faustovirus particles and VFs in different stages of the replication cycle (4). Although cysts not containing viral particles or VFs are able to excyst (7), cysts containing faustovirus particles and VFs do not have the ability to excyst (8). In addition to viruses, VFs are trapped inside the cyst (9), hampering faustovirus mariensis dissemination. Viral factory: VF

A recent study described a new antiviral mechanism employed by the host amoeba *V. vermiformis* to evade infection by faustovirus mariensis [68]. Faustovirus mariensis is a strain isolated from water samples in Brazil. The genome of the virus is composed of a circular, double-stranded DNA molecule, approximately 460 kb, surrounded by an icosahedral capsid with a size of approximately 190 nm [68]. The first faustoviruses strains were isolated from *V. vermiformis* in France and Senegal [8]. As described for other faustoviruses isolates, faustovirus mariensis infects *V. vermiformis*, inducing cell lysis (Fig. 3). In addition, it has been demonstrated that faustovirus mariensis is able to induce the formation of plaque-forming units, and lysis of the host cell is an essential way for efficient dissemination of faustovirus particles.

During the replication cycle study of faustovirus mariensis, was observed elevated formation of *V. vermiformis* cysts, unlike those observed in *V. vermiformis*, infected by other giant viruses such as tupanvirus and orpheovirus. Curiously, faustovirus mariensis particles, as well as distinct phases of its replication cycle, were observed inside the cytoplasm of *V. vermiformis* cysts. It was demonstrated that the formation of *V. vermiformis* cysts during faustovirus mariensis infection occurs in a MOI-dependent way, wherein at high MOIs almost all *V. vermiformis* trophozoites were converted to cysts. In addition, low MOIs were associated with viral replication, while higher MOIs were associated with lower rates of viral multiplication. These observations suggested that the virus was able to infect the host cell, but it was not able to release its progeny since particles and VF were retained inside the cysts (Fig. 3).

As described in Section 1 of this review, the expression of cellular serine proteinases is related to the encystment process, and the regulation of one of these enzymes by mimivirus is associated with inhibition of the encystment process in *A. castellanii*. Since mimivirus is only able to replicate in trophozoites and not in cysts, prevention of encystment is critical for the replication of this virus. Faustovirus mariensis, on the other hand, was not able to block the *V. vermiformis* encystment, and at high MOI, trophozoites were converted to cysts and viral replication was not observed. Additionally, faustovirus mariensis induced the expression of serine proteinase present in *V. vermiformis*, suggesting that this virus is not able to regulate one of the factors that trigger the encystment of *V. vermiformis*. Finally, it was shown that the inoculation of fresh *V. vermiformis* trophozoites using the supernatant of infected *V. vermiformis* cultures can induce encystment in a dose-dependent way, suggesting that trophozoites infected by faustovirus mariensis release factors that can trigger encystment (Fig. 3).

The release of soluble factors has already been associated with the encystment process in *A. castellanii* [35]. Furthermore, search for the nature of the factors involved in this phenomenon revealed that encystment factor(s) were likely not proteins since treatment with proteinase K or bromelain was not able to prevent the encystment of *V. vermiformis*. It was through measurement of the different inorganic factors in the supernatants of faustovirus mariensis-infected cells compared to a giant virus that does not induce encystment (tupanvirus), which made it possible to suggest one of the factors responsible for the induction of the encystment in this system. Based on these findings and a previous study showing that Mg^2+^ is a factor that triggers encystment in *A. castellanii*, we tested the potential of Mg^2+^ to trigger the encystment of *V. vermiformis* [68, 69]. It was observed that magnesium-ion input not only stimulated encystment, but it also promoted a gradual increase in Mg^2+^ concentration in the supernatant of cells, which can act as an encystment stimulus for neighbor trophozoites. We also observed that ethylenediaminetetraacetic acid (EDTA) (a bivalent cation inhibitor) affects encystment factor activity, reinforcing the importance of Mg^2+^ in cell communication, in this context [68].

Although previous studies have demonstrated that intracellular bacteria, such as *Salmonella enterica* and *Escherichia coli*, are able to survive and take advantage of amoebal encystment. This was the first study to demonstrate the entrapment of viral particles and VF inside amoeba cysts [68, 70]. In addition, evolutionary issues derived from this interaction appear to be unique since amoeba cysts containing bacteria are able to excyst returning bacteria to multiply. This is not observed for amoeba cysts containing faustovirus. The study revealed that only cysts without faustovirus mariensis in their cytoplasm were able to excyst. Thus, the interaction between faustovirus mariensis and the encystment of *V. vermiformis* was suggested as a novel type of antiviral strategy, in which faustovirus mariensis dissemination is hampered (Fig. 3). Analogously, this mechanism was associated with the antiviral interferon system in vertebrates [68].

### One more member in the giant virus–host interactions: the virophage

The study of giant viruses has become even more complex due to the discovery of small viruses capable of infecting them, such as the virophage. The first virophage, called sputnik, is about 50 nm in size and approximately 18 kbp, with circular double-stranded DNA, and it was found to be associated with a strain of mimivirus [15]. The virophages are unable to multiply in the absence of giant viruses. Their replication occurs in the giant virus factory and can be deleterious to viral replication, resulting in a decrease in amoebae lysis [15, 71]*.* Since their discovery, dozens of new virophages have been isolated and classified in a new viral family called *Lavidaviridae* [72–80]. It is believed that the virophage can mediate lateral gene transfer between giant viruses. Furthermore, they have been shown to be able to integrate into giant viruses and host cell genomes. These findings strongly suggest that amoeba, virophages, and giant viruses seem to co-evolve with each other [15, 81, 82]. The discovery of new virophages led to the description of some interesting interactions between virophages, giant virus and hosts. In 2014, a virophage named zamilon was isolated, which, unlike the virophages described to date, was not able to replicate in factories of mimiviruses from lineages A, but only in mimivirus factories from lineages B and C [76]. Its host specificity aroused the curiosity of Levasseur and collaborators, who studied the genetic basis of this host specificity [83]. It was observed that strains of the mimivirus lineage A, resistant to the zamilon virophage, contain the insertion of a repeated zamilon sequence in its genome. These repetitions were named mimivirus virophage resistance elements (MIMIVIREs). By analyses of the surrounding sequences the authors observed that the MIMIVIRE system presents nuclease and helicase proteins, which may play a vital role in the degradation of foreign nucleic acids, suggesting that this locus can be related to the clustered regularly interspaced short palindromic repeat (CRISPR)-Cas system, although it is not homologous to this system [84]. Interestingly, the silencing of the MIMIVIRE genes restored zamilon’s ability to infect the factories of mimivirus lineage A. As a result of which, the researchers proposed that the MIMIVIRE system acts as a viral defense mechanism against virophages [83]. Recently, additional biological demonstrations enabled further characterization of the MIMIVIRE system defense mechanism. It was demonstrated that a mimivirus gene of unknown function, called R349, one of the MIMIVIRE system components that contains four repeats homologous to the virophage sequence, has a key function in the MIMIVIRE system defense mechanism. The deletion of the R349 gene in mimivirus lineage A restored the replication of zamilon. In addition, it was observed that a mimivirus isolate of lineage A, lacking 3 of 4 repeats of R349 gene, was susceptible to zamilon infection [85]. Considering the above mentioned, these results reinforce the role of the MIMIVIRE as a nucleic-acid-based immunity defense system against virophage infection, confirming the importance of the R349 gene in the MIMIVIRE system. This study revealed an unprecedented type of host–virus interaction and reinforced that host amoeba, virophages, and giant viruses are coevolving. Another notable virophage–giant virus–host interaction is that which involves the marine protist *Cafeteria roenbergensis* with the C. roenbergensis giant virus and its associated virophage, mavirus. Cafeteria roenbergensis virus (CroV) is distantly linked to mimiviruses that infect the phagotrophic biflagellate *Cafeteria roenbergensis* [72]. Mavirus was the second virophage discovered, isolated from water collected in Texas, USA [73]. The mavirus virophage replicates in the viral factory of CroV; however, it was observed that the mavirus can enter into *C. roenbergensis* independent of CroV by endocytosis and is able to inhibit the production of new CroV particles, increasing the survival of the host *C roenbergensis* [73]. In 2016, Fischer and Hackl discovered through the co-infection of a host population with CroV and mavirus that the virophage is able to integrate into the genome of *C. roenbergensis* [86]. They showed that the mavirus genome was integrated at different genome locations, and although the integrated virophage genes are not constitutively expressed, they can be activated by CroV infection, inducing the production of infectious mavirus particles and reactivating this virophage in the host cell. Although this was expected, the reactivation of the mavirus was not able to block the replication of CroV, and, consequently, *C. roenbergensis* infected with CroV died anyway, releasing CroV and mavirus particles. In spite of this, they observed that the released mavirus decreased the spread of CroV in the protist population and its replication in another replication cycle, protecting the neighboring cells from being killed by the giant virus infection. The authors associated this virophage–giant virus–host interaction as an altruistic defense mechanism of the host, in which a host dies, releasing viral particles that are able to protect the neighboring host population [86]. Another possibility is that this interaction acts as an adaptive immunity CRISPR-Cas system, in which the virophage genome is retained by the host and used to prevent subsequent attacks by the giant virus. Viral elements can be found in eukaryotic genomes; however, little is known about how they act and their functions [87]. This study provided an example of a virophage that integrates into a cell genome, acting as an inducible antiviral defense system. It has been demonstrated that a green alga called *Bigelowiella natans* contains virophages integrated into its genome, providing another possible example of a virophage-mediated host defense [82]. In addition to these virophage integration studies, several peculiarities have been observed in the virophage–giant virus–host interactions. Among these was a study showing that the virophage sputnik and marseillevirus co-infection affected the replicative capacity of marseillevirus [88]. Using a metagenomic approach, it was suggested that virophages reduce the mortality caused by the giant viruses of phototrophic algae, and through the use of a mathematical model, it was proposed that besides the direct interference in the multiplication of giant viruses, virophage infection can select viruses with reduced replicative capacity, contributing to the protection of the host cell population [74, 89]. Based on this and other studies, it has been suggested that virophages are associate with the regulation of the population of amoebae and other protists in the environment [90]. In 2018, a virophage was isolated and said to be associated with a mimivirus strain that infects *Saccamoeba* spp., with the ability to induce a high reduction (~ 70%) in viral capsid production [91]. The growing description of new virophage isolates and new interactions involving them has revealed that virophages, giant viruses and its, host make up a complex and unprecedented type of host–virus interaction and that there are probably still many interactions to be studied.

## Conclusions

Giant viruses have surprised us, not only with respect to their genomic and structural complexity, but also due to groundbreaking findings showing their ability to establish intriguing host–pathogen interactions. Although many studies involving giant viruses have been published in recent years, most of them have been focused on new virus discovery and evolution, and the molecular aspects of giant virus–host interactions remains largely unknown [3–12]. Giant virus characterization studies have revealed a potential of future surprises in giant virus–host interactions. Evidence of this potential is that giant viruses have been found in diverse and unexplored environments, where they may be interacting with more organisms than we can imagine [14, 29, 92, 93]. Sequences of several giant viruses were found in human microbiome, but nothing is known about their interaction profile and ecological roles [94, 95]. Furthermore, it has been found that these viruses can encode genes that act on complex biochemical pathways [96–98]. The wide distribution and diversity of giant viruses associated with their powerful gene arsenal, both known and unknown, can reflect the wide range of interaction strategies. Although most discovered giant viruses are associated with amoebae, the spectrum of giant virus hosts may be larger than what has been discovered so far. The future expansion in the isolation culture methods may bring surprises in relation to giant viruses associated with other host types, which also broadens the possibilities for virus–host interactions studies [28]. Besides that, the metatranscriptomic may reveal novelties in the study of giant virus interactions, as a method that does not require the culture of organisms, a challenge often encountered in establishing virus–host interactions. A study using this approach suggested that previously unknown virus–host relationships in marine systems are abundant [99]. Although biological confirmation of findings and validations of host–virus interaction studies in natural microbial communities is important, metatranscriptome-based studies can point to new findings involving organisms that cannot be grown in cultures. These and other reasons make future studies involving giant virus–host interactions challenging, and although there has been impressive progress in the giant viruses field, the study of giant viruses is new and there is still much to learn about their host interactions and ecological roles.

## Data Availability

Data sharing not applicable to this article as no datasets were analyzed or generated during the current study.

## Abbreviations

APMV: Acanthamoeba polyphaga mimivirus
CPE: Cytopathic effect
CRISPR: Clustered regularly interspaced short palindromic repeat
CroV: Cafeteria roenbergensis virus
EDTA: Ethylenediaminetetraacetic acid
EMSP: Encystment-mediating subtilisin-like serine proteinase
ER: Endoplasmic reticulum
MBP: Mannose-binding protein
MIMIVIRE: Mimivirus virophage resistance element
MOI: Multiplicity of infection
MsV: Marseillevirus marseillevirus
VF: Viral factory
